# Dating Violence and the Quality of Relationships through Adolescence: A Longitudinal Latent Class Study

**DOI:** 10.3390/bs14100948

**Published:** 2024-10-15

**Authors:** Carmen Viejo, Rosario Ortega-Ruiz, María Sánchez-Zafra

**Affiliations:** Department of Psychology, Faculty of Educational Sciences and Psychology, University of Cordoba, 14071 Cordoba, Spain; cviejo@uco.es (C.V.); ed1orrur@uco.es (R.O.-R.)

**Keywords:** adolescence, dating violence, violent profiles, relationship quality, latent class analysis, longitudinal study

## Abstract

Dating violence can manifest itself in different ways, with important consequences for both members of the couple. Due to the normalization of certain behaviour and the perceived quality of the relationship, it may be difficult to identify this violent dynamic in its early stages, allowing it to escalate and lead to severe levels of violent behaviour. This study aims to analyse violent profiles using latent class analysis (LCA) and explore the role of relationship quality variables as risk factors for those profiles. A two-stage longitudinal design was used with a sample of 2849 Spanish adolescents between 12 and 18 years old. The LCA produced five different groups: those not involved in violence; those involved in mild forms of violence (with two sub-groups: those involved in psychological–sexual violence and in psychological–physical violence); and those involved in the most severe forms of violence, with a co-occurrence of psychological–physical–sexual violence (with two sub-groups, according to the higher of lower frequency of involvement). Despite the differences between boys and girls, negative-quality variables played a more important role in dating violence. Discussion of the results focuses on both the complex nature of the violence and the multi-probabilistic view of its development.

## 1. Introduction

Young people begin to show interest in the processes of courtship and mutual choice that lead to their first romantic relationships from early through to late adolescence (between the ages of 10 and 18), and at some time in this developmental period, the majority of boys and girls experience their first romantic relationships [1]. This moment is a key psycho-developmental milestone, involving, to a large measure, the adolescent’s social identity, especially with regard to their social competence and the management of their emotional life [2]. A kind of mechanism emerges at this point in which the new emotions that these experiences generate stretch to the utmost the few competencies and skills which adolescents have to face these challenges. In this way, adolescents try different, variated strategies that do not contribute to an optimal emotional balance within the affective framework of an intense love affair. Adolescents usually perceive the romantic situation as highly demanding, and the strategies and attitudes that they use to cope with it are felt to be inadequate to the demands of the situation from a dynamic perspective [3]. All of this can lead to possible conflict, increased pressure, and, finally, in some cases, inappropriate behaviour and violence [4].

Dating violence is defined as a phenomenon featuring aggressive behaviour which occurs in relationships between adolescent couples, regardless of the stability or duration of the relationship, which these days is often short and unstable [5]. It is characterized by a sporadic pattern of low-intensity, reciprocal, and bidirectional aggressive behaviour, in which both members of the couple assume roles as aggressors and victims [6]. This bidirectional pattern of aggression–victimization can shift during the relationship, with increased intensity and the use of different forms of violence (psychological, physical, and/or sexual), and the roles can be polarized to include an aggressor and a clearly defined victim [7,8]. Some studies have shown that this development of the dynamics of violence in the relationship occurs progressively, with different types of psychological violence occurring first (coercion, humiliation, detachment, etc.). As the relationship progresses, and this behaviour becomes established as part of the relationship dynamic, other types of violence such as physical and/or sexual behaviour can occur. There seems to be a progression in violence, like an escalation, from the mildest violent behaviours to a co-occurrence of different forms of aggression, with varying degrees of intensity and sometimes distinct roles of involvement [9].

The different patterns of participation in this form of violence have made it difficult to obtain homogeneous data about involvement in the phenomenon. Many of the studies which have tried to analyse this problem have done so from an individualized approach to each form of violence, without taking into account the co-occurrence of behaviour or the different forms of involvement in violence. It has been found that rates of victimization in physical violence range between 0.4% and 53.7% in boys and between 1.2% and 41.2% in girls [10], while the rates of perpetration of sexual violence range between 2.6% and 58.8% in boys and between 1.2% and 40.1% in girls. Meanwhile, sexual violence victimization rates range between 0.1% and 54.2% in boys and between 1.2% and 64.6% in girls [11]. The most recent meta-analyses estimate a global involvement that ranges between 6% and 37%, with gender differences depending on the type of violence: perpetration of psychological violence (verbal–emotional) ranges between 4.3% and 95.3% in boys and between 4.2% and 97% in girls [12]. However, couple relationships usually involve various types of violence which are manifested in both mild and complex ways, and few studies have analysed the implications of this co-occurrence.

In order to move towards the characteristics that make up the phenomenon, the latent profile analyses provide an approach of involvement according to the individual specific pattern of violent behaviours, offering a more complex, nuanced understanding [13,14]. Some studies which have adopted this study approach have identified between three and five profiles or categories depending on how these underlying patterns are articulated and the relational dynamics they include [15,16]. These studies often reveal profiles with low levels of involvement in the different forms of violence and others with high levels of co-occurrence, while the rest of the profiles usually vary depending on whether aggression and victimization are analysed individually or both together. Bidirectionality is a key characteristic of this kind of violence, and it is therefore necessary to carry out analyses of risk profiles with aggression and victimization together, assessing the variability of the groups of involvement and exploring the holistic understanding of the phenomenon.

### 1.1. Risk Factors for Involvement in Violence: The Quality of the Relationship

It is equally important to identify which variables could be behind this progressive pattern of violence, making the behaviour more complex and intense as the relationship progresses, and which variables, in contrast, help individuals to manage it appropriately and achieve a good dynamic in the relationship or, in some cases, the breakup. The literature has identified three large groups of factors that could constitute the explanatory basis for the appearance of this violence: individual factors, contextual factors, and factors inherent to the couple’s relationship itself. Nonetheless, the previous involvement in any form of violence within the couple context has been demonstrated as the main risk factors to evolve in the violent dynamic [17].

If we consider individual factors, attributes such as age, ethnicity, gender, socioeconomic status, levels of self-esteem, anger, hostility, depression, substance abuse, low tolerance for frustration, and hostile beliefs and attitudes are all considered risk factors for involvement in violence [18]. Gender differences in perpetration and victimization rates remain a controversial issue, although it is notable that there is a higher risk of victimization in girls than in boys [19,20].

In the same way, it has been shown that contextual variables, particularly those related to the social environment, such as previous experiences of victimization or the development of insecure attachment styles to significant figures, contribute directly to the onset of this phenomenon. In this sphere, stereotypes and the normalization of social behaviour in line with the myths of romantic love and the prevailing sexist beliefs have been found to be relevant indicators [21].

In regard to the dynamics of the romantic relationship itself, the duration of the relationship, levels of satisfaction, and the conflict resolution strategies used have been shown to be predictors of involvement in violence [22]. In contrast, high levels of well-being both in the individuals and in the relationship have been cited as protective factors against such involvement [23], along with factors such as the positive quality of the relationship and competence in conflict resolution. In this context, the quality of a relationship probably seems to be the main determining factor in the initiation and perpetuation of the phenomenon of dating violence [24,25]. A higher-quality relationship appears to act as a protective factor, lowering the likelihood of involvement in violent behaviour.

Relationship quality is defined as a measure of the degree to which a relationship meets the expectations, needs, and desires of the individuals involved. The construct of the quality of the relationship is evident in positive variables such as satisfaction with the relationship, approval, and commitment and in negative variables such as conflict, domination, pressure, and criticism [26,27]. However, the perception of the quality of the relationship is always a subjective value and depends on compliance with the intrapersonal and interpersonal variables formed by the individuals involved [28]. Relationships perceived to have high levels of positive-quality traits correlate with greater satisfaction, well-being, affection, intimacy, trust, security, mutual care, and emotional support, while those perceived as low quality present high rates of conflict and antagonism [29,30]. In addition, positive-quality characteristics are associated with a more positive mood and higher overall personal well-being [31]. In contrast, relationships marked by low satisfaction are linked to depressive behaviour, anxiety, learning difficulties, etc., which can lead to the appearance of, or further perpetuate, violent behaviour [32,33]. Its role in the violence escalation dynamic would be crucial. Low quality has been demonstrated to be linked to higher rates of unresolved conflicts, and they are related to different violent behaviours. Partners may use violence as a way of expressing frustration, exerting control, or managing stress within the relationship, evolving in the escalation of a violent dynamic.

### 1.2. The Present Study

A lower quality of the relationship can increase the risk and facilitate the appearance of violence in the couple, especially with psychological violence [34]. For this reason, the present article tries to focus on analysing relationship quality as a possible predictor of dating violence, exploring how the different manifestations of this construct influence the emergence and perpetuation of these behaviours. The scientific literature has pointed out the relationship between quality and violence [4,30,31] but has not approached this study from a longitudinal perspective that would allow us to determine the weight it has as a risk factor.

This study follows two main aims: (1) examine the different profiles or types of involvement in violence by identifying hidden heterogeneous groups (latent classes) based on patterns of bidirectional involvement in intimate partner violence (taking into account the type of violence and its frequency). In line with previous research [34], we have put forward the hypothesis that there are at least three coexisting groups or profiles, according to the types of behaviour employed and taking into account the pattern of the progression of violence; (2) examine the relationship of these groups or classes with variables related to the quality of the couple, analysing whether the variables of relationship quality are part of the predictor factors for belonging to one category or another. We have hypothesized that positive-quality variables may be having a greater casual weight as protective factors and negative-quality variables as risk factors for involvement in violence [23,32,33].

## 2. Materials and Methods

### 2.1. Participants

A total of 2849 adolescents between 12 and 18 years old, attending schools in the Autonomous Community of Andalusia (Spain), took part in this study. To achieve the objectives proposed, we chose boys and girls who had or had had romantic experiences during the entire study period. The final sample consisted of a total of 575 children (58.1% girls; 40.0% boys; and 0.5% others), with an average age of 14.75 (S.D. = 1.81).

### 2.2. Instruments

We used two self-report instruments with suitable psychometric properties to measure violence in romantic relationships and their quality:

*The Revised Dating Violence Questionnaire* [35,36] is the short version of the original [37], an instrument that measures aggression and victimization with a romantic partner in five dimensions: coercion (α = 0.89 in aggression; α= 0.88 in victimization, e.g., you have/they held him/her/you back from leaving); sexual violence (α = 0.82 in aggression; α = 0.83 in victimization, e.g., you force your partner/you feel obliged to have sex even when you/they does not seem to feel like it); physical violence (α = 0.96 in aggression; α = 0.92 in victimization, e.g., you have/they have slapped, pushed, or shook him/her/you); detachment (α = 0.87 in aggression; α = 0.89 in victimization, e.g., you are/they are a good student but are always late for meetings, do not fulfil your/his/her promises, and are irresponsible); and humiliation (α = 0.95 in aggression; α = 0.97 in victimization, e.g., you/they ridicule his/her/your way of expressing him/herself/yourself). This version consists of 20 items scored on a 5-point Likert-type scale (0—never to 4—all the time).

The quality of the relationship was measured using the *Network of Relationships Inventory—Relationship Qualities version* [38] (NRI-RQV, Buhrmester and Furman [38]), a 30-item scale that measures the quality of relationships using a 5-point Likert-type scale (—never or hardly ever to 4—always). It evaluates five positive characteristics, including companionship (α = 0.83, e.g., How often do you spend fun time with this person?), intimate communication (α = 0.90, e.g., How often do you tell this person things that you do not want others to know?), emotional support (α = 0.85, e.g., How often do you turn to this person for support with personal problems?), approval (α = 0.90, e.g., How often does this person praise you for the kind of person you are?), and satisfaction (α = 0.89, e.g., How happy are you with your relationship with this person?), and five negative relationship characteristics, including conflict (α = 0.90, e.g., How often do you and this person disagree and quarrel with each other?), criticism (α = 0.92, e.g., How often does this person point out your faults or put you down?), pressure (α = 0.88, e.g., How often does this person push you to do things that you do not want to do?), exclusion (α = 0.87, e.g., How often does this person not include you in activities?), and dominance (α = 0.93, e.g., How often does this person get their way when you two do not agree about what to do?).

### 2.3. Procedure

We carried out a prospective longitudinal study with two waves six months apart (October 2022–April 2023). The study was approved by the University of Córdoba Bioethics and Biosafety Committee, and informed consent was obtained from the heads of the participating schools, the families, and the participants themselves. The two data collections were administered during school hours, and the anonymity of the data and the voluntary nature of participation were guaranteed at all times. Each student was assigned a code, which enabled us to link their first and second questionnaires. Each questionnaire took around 50 min to complete.

### 2.4. Data Analysis

We used a two-time data matrix for all the relevant variables. The data were subjected to rigorous follow-ups to mitigate the attrition rate, analyse missing data, adjust for confounding variables, and ensure the consistency of instruments and data collection methods. Latent class analyses (LCAs) were performed using MPLUS 8 [39]. Using this analysis, we obtained underlying categories, by which we grouped individuals according to similar patterns in their responses or characteristics. To carry out the LCA, we took the variables of involvement in violence from the second data collection as a reference, with the data obtained from the participants based on their responses in different subscales: aggression and victimization in psychological violence, aggression and victimization in physical violence, and aggression and victimization in sexual violence. The optimal number of classes resulted from the interpretability of the classes and the following criteria: Bayesian (BIC), likelihood ratio test (LMR-A), and entropy. The model with the lowest values in BIC [40] and an entropy value greater than 0.80 and closer to 1 [41] was considered the best solution. Next, we generated an independent matrix to include the results of the latent classes and performed Multinomial Regression Analyses differentiated by sex to identify the role of quality in the involvement in violence. Student’s *t*-test was conducted to check the differences between the responses of the boys and girls. Considering the results of the latent classes as the dependent variable, the different positive- and negative-quality variables obtained in the first data collection were taken as independent variables; that is, the quality measure was taken six months before measuring the involvement in violence. The descriptive analyses were carried out using SPSS version 11.

## 3. Results

### 3.1. Latent Classes of Involvement in Violence

We carried out an LCA to identify groups of adolescents in relation to their responses regarding involvement in aggression and victimization in violent behaviour in the context of couples.

Four models were estimated, using between 2 and 5 classes. Table 1 shows the goodness-of-fit indices of each model. The AIC and BIC values decreased from the two-class solution to the five-class solution. The five-profile solution was considered the most appropriate, obtaining the lowest values of these fit indices and a satisfactory entropy value, which suggests that this solution achieved the best degree of classification.

**Figure 1:**
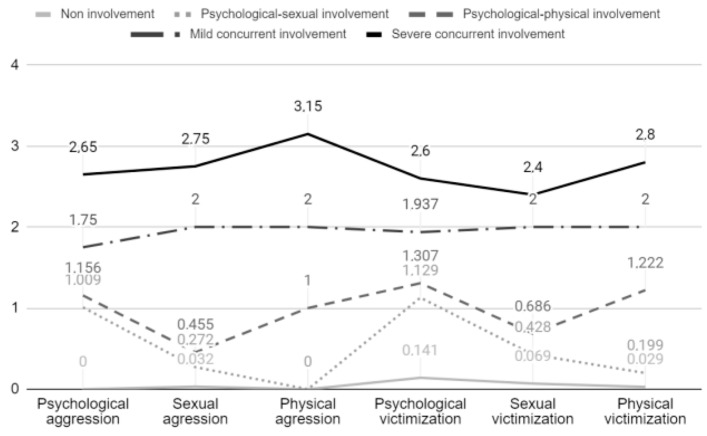
Description of the 5 categories. Average involvement in the different types of violence considered. Note: Scores range from 0 (I have NEVER perpetrated/suffered this behaviour) to 4 (I perpetrate/suffer this behaviour ALL THE TIME).

The five-class model allowed us to differentiate between the following groups of involvement: the first group (*n* = 406) showed scores of near to zero in terms of aggression in the three types of violence, low levels of victimization in psychological violence (0.14), and very low levels of sexual (0.07) and physical violence (0.03). For these characteristics, it was labelled the “non-involvement” group. The second category (*n* = 102) presented the highest scores in involvement in psychological and sexual violence, with medium–low values in both aggression (1.01 and 0.27, respectively) and victimization (1.13 and 0.43, respectively) and was labelled the “psychological–sexual involvement” group. The third category (*n* = 31) presented significantly higher rates of psychological and physical violence, both in aggression (1.16 and 1.00, respectively) and in victimization (1.21 and 1.22, respectively) and was labelled the “psychological–physical involvement” group. The fourth category (*n* = 20) was termed the “mild concurrent involvement in violence” group and was characterized by high mean scores in aggression (between 1.75 and 2.00) and victimization (between 1.94 and 2.00) in all types of violence. The last group (*n* = 16) was analogous to the previous one, but with even higher scores (between 2.65 and 3.15 in aggression and between 2.40 and 2.80 in victimization) and was labelled the “severe concurrent involvement in violence” group (see Figure 1).

According to this five-latent class model, 70.60% of the participants were not involved in any forms of violence. Considering an escalation model of violence, 23.10% of participants were occasionally involved in milder bidirectional forms of violence, which included the co-occurrence of psychological–sexual violence (17.70% involvement) and psychological–physical violence (5.40% involvement). Moving up to higher levels, 3.50% of the participants reported involvement in occasional forms of violence, including psychological, physical, and sexual behaviour. Finally, 2.80% of the participants admitted to being involved in more frequent violent behaviour, including psychological, physical, and sexual forms of violence.

### 3.2. Relationship Quality and Involvement in Violence

Multinomial regressions analyses were performed, taking those of negative and positive quality as independent variables and the latent classes of involvement in violence as dependent variables.

Independent samples *t*-test difference analyses were also performed to compare the scores on the positive- and negative-quality variables between boys and girls, thus determining whether the regression models should be grouped or differentiated. Significant differences were found in the positive quality of commitment (t (567) = 0.116, *p* < 0.05, d = −0.207); emotional support (t (567) = 0.097, *p* < 0.05, d = −0.0.209); and approval (t (567) = 0.076, *p* < 0.05, d = −0.268), with girls scoring higher in all cases. Similarly, among the negative-quality variables, we found significant differences in conflict (t (567) = 1.875, *p* < 0.05, d = 0.249); criticism (t (567) = 25.155, *p* = 0.000, d = 0.568); pressure (t (567) = 39.934, *p* = 0.000, d = 0.677); exclusion (t (567) = 21.761, *p* = 0.000, d = 0.475); and domain (t (567) = 9.948, *p* = 0.000, d = 0.74), with the boys scoring higher in all the variables. These results suggest that creating differentiated models for boys and girls was the correct approach.

To interpret the results of the impact of quality on violence, we considered the violence groups in terms of their escalation in behaviour, assessing the role of the quality of the relationship in the risk of this escalation.

First, we estimated the sample sizes of each of the relevant profiles independently for boys and girls (see Table 2).

Since some of the involvement groups showed a very low frequency, we considered redefining them in three violence groups according to an escalation of the behaviour, so the “psychological–sexual involvement” and “psychological–physical involvement” groups were joined into one group labelled “separate involvement”, and the “mild concurrence involvement” and “severe concurrence involvement” were combined into a group called “concurrent involvement”. The analyses were then carried out on the three resulting groups: non-involvement, separate involvement, and concurrent involvement.

Firstly, we considered the role of quality as a risk factor to advance from non-involvement to separate involvement (see Table 3).

The boys’ regression models indicated that the conflict variable (β = 0.616, OR = 1.85, 95% CI [1.12, 2.81], *p* < 0.05) acted to increase the risk of involvement in violence.

For girls, the conflict variable increased the risk of involvement in violence (β = 0.529, OR = 0.486, 95% CI [0.264, 0.897], *p* < 0.05), while the critical variable (β = −0.721, OR =1.697, 95% CI [1.20, 2.38], *p* < 0.05) decreased the risk of involvement.

The following regression model assessed the impact of quality as a risk factor for progressing towards a more severe form of violence involving the concurrence of psychological, physical, and sexual behaviour (see Table 4).

The models used for boys showed that the conflict (β = 0.710, OR = 0.492, 95% CI [0.246, 0.985] *p* < 0.05) and pressure variables (β = −0.810, OR = 2.249, 95% CI [1.073, 4.711], *p* < 0.05) increased the risk of progressing towards concurrent violent behaviours. In contrast, the emotional support variable reduced the risk of advancing from separate to concurrent violence (β = −0.841, OR = 0.431, 95% CI [0.198, 0.940], *p* < 0.05).

Among girls, no significant differences were found between the quality variables that increased or decreased the risk of advancing from separate forms of violence to concurrent violence.

## 4. Discussion, Conclusions, and New Research Challenges

The first objective of this work was to analyse the different profiles of involvement in violence in adolescent couples, given that there is still little research available which offers an integrated approach to involvement in this phenomenon, and that the rates of involvement recorded so far may even be underestimated, as they consider each form of violence in a differentiated way.

According to the research, a holistic view of this violence has identified between three and five differentiated involvement profiles or groups. Our results point in the same direction, with a solution of five latent classes, which reflect, to a large extent, a progressive escalation of violence [9]. The results reveal four different profiles of involvement in patterns of medium–high frequency violence, corresponding to 29.4% of the sample, and a fifth group, the largest group, corresponding to boys and girls who are not involved in any form of violence in their relationships. These findings are consistent with other similar studies [15,16] which found very similar profiles to those in our study.

The analysis of the four groups involved in violence allows us to identify the different moments of severity in violent behaviour. From the most subtle to the most severe forms, boys and girls involved in violence in their relationships showed similar rates of aggression and victimization, thus reflecting the bidirectional nature of violent behaviour in adolescent romantic relationships [8]. A considerable number of boys and girls are involved in occasional forms of violence, including psychological–sexual or psychological–physical violence. This group can be considered to be those involved in low-intensity violence in its most subtle forms.

In the second stage of the escalation in violence, the results point towards a concurrence of violent behaviour, occurring occasionally at first, and then with greater frequency. Several studies have pointed to the normalization and acceptance of violent behaviour as a key factor in this escalation [21]. The presence of psychological forms of violence together with other forms of violent behaviour from the very first stages of violent behaviour appears to be in line with the results that point to the early appearance of psychological violence as a precedent for other types of violence, such as sexual or physical [42]. Here, the acceptance or normalization of psychological violence in a relationship could act as a significant risk factor for the subsequent development of other types of violence among adolescents [43]. However, to obtain conclusive results in this area, studies would be needed which examine the transition over time in these latent classes, and how they evolve in the dynamic context of adolescent couple relationships, considering the sample size of the groups involved in more severe violence.

Likewise, there is a clear differentiation between the two well-identified groups which alternatively use physical violence and sexual violence, an aspect which has already been identified in the previous literature [44,45]. Some authors [46,47] have shown that, although sexual violence can be treated as an added element in other types of violence (verbal, physical, etc.), it can also be understood as a form of violence per se, with well-differentiated characteristics and development patterns which form their own trajectories of involvement [48]. Therefore, in adolescence, a developmental stage in which sexual development is at the forefront, the rates of involvement in sexual violence could depend on how they manage certain erotic–sexual behaviour, which can be irritating, not be desired equally by both members of the couple, or not be accepted because it is not managed properly [5].

The important causal role of relationship quality in the involvement in teen dating violence has been demonstrated. According to previous studies, it acts as both a protective and a risk factor [4]. In this study, we examined relationship quality prior to latent class analysis, thus fulfilling the second aim of the study. Latent class analysis of violence involvement and its relationship-to-relationship quality as a possible risk factor can provide a valuable input for the design of specific prevention programmes tailored to the profile of those involved. However, the present analyses do not determine the evidence for relationship quality as a predictor of intimate partner violence. The results showed the importance of the causal effects of relationship quality, and involvement in violence six months later, with significant differences between boys and girls. According to the reference literature, the importance of quality is greater in the first stages of a violent escalation than in the later phases, probably because the greatest risk factor for moving towards more severe forms of violence is the person’s previous involvement in milder forms of violence [49].

In this context, despite the differences between boys and girls, negative quality plays a relevant causal weight as a risk factor. In both, conflict seems to be a key variable for the appearance and progression of violence, which has already been identified by other authors [50]. Similarly, pressure increases the risk for boys of moving towards involvement in more severe violence. Here, the previous literature has pointed out that negative-quality variables are risk factors for the development of violent escalation [33].

In contrast, positive quality plays a more discrete role. Only in the case of boys has the variable of positive quality of emotional support been identified as a strong causal protective factor against progression to more severe violence. This finding suggests that work on these variables could help to enhance the competencies needed to prevent violence [51,52], although it does not play a leading role, so it is important to analyse the role of these variables as mediators, or as moderators of the direct effects of other variables, considering the differences between boys and girls as well.

In the same way, it is important to look at the differences between boys and girls, as models for girls are more complex and produce fewer results than those for boys. Although the literature has already highlighted the complexity underlying the phenomenon of violence in girls, interpreting it in a rather more confusing and diffuse way [53], we need to make progress in the specific study of this population due to the devastating consequences that have been identified in girls [4]. This study was limited by the small sample available for the groups involved in the most severe violence, which limited the type of analysis and the results obtained. The normalization of certain behaviour may make it difficult to identify this problem among younger adolescents. Further studies are needed along these lines to clarify the role of these variables, particularly in the dynamics of violence among girls. Another important limitation of this study focuses on the statistical technique provided. Although it did not correspond to the objective of the study, a latent transition analysis in two stages, and its relationship with the quality of the relationship, would have provided the study with the ability to specify the importance of the quality of the relationship in the development of violence. This will be taken into account for future research that has different objectives.

Despite these limitations, this longitudinal study, which examines the relationship between quality variables and various latent violence risk profiles six months later, offers interesting and innovative results which could help us understand the complexity of these psycho-developmental processes. They are largely characterized by the incompetence and lack of expertise in emotional relationships which the protagonists find extremely demanding and which they have to face without any experience or true understanding of the meaning of this new model of emotional life. Furthermore, our results give us an important insight into how to address violence prevention practises in adolescent couples, as well as advice for when new romantic couples are formed in the future.

The first main point is the significant role played by psychological violence in the spectrum of violent behaviour, as it is present in all the identified risk profiles. This form of violence can act as a precursor or facilitator of other types of violence, thus justifying its inclusion in preventive strategies that seek to interrupt the progression towards more severe forms of aggression.

This study has identified four different risk profiles that seem to point to an increasing scale of risk of violence, and one of the key ways of stopping this escalation could be to identify the first signs of violence, and to avoid the normalization and acceptance of such behaviour.

As an innovation with respect to other related studies, this study also reveals significant gender differences in the relationship between quality variables and the probability of involvement in violence. For both boys and girls, negative-quality variables, in particular conflicts, act as a risk factor for the development of violence in the adolescent couple, thus revealing their key role in involvement. This analysis supports the relevance of the influence of relational quality in the prevention of intimate partner violence, which is well documented in the literature.

The transition from milder violence to more severe violence in girls remains a matter of controversy, and future lines of research are needed to explore this field further.

The nature and scope of the study are of great importance in the Spanish context. In Spain, there is little research on this subject. The results on the existence of different profiles and gender differentiation differ depending on the context. In this case, the present research draws a framework of characteristics in this respect focused on the Spanish context, which can be used to work on them in prevention and intervention programmes adapted to Spanish boys/girls.

The design of a longitudinal study with two waves, together with the measurement of quality variables as risk/protective factors six months before the LCA, has allowed us to establish patterns and causal relationships between the quality of the relationship and the manifestation of violence. This methodology provides a solid foundation for early interventions and preventive strategies focused on strengthening the quality of adolescent relationships.

## Figures and Tables

**Table 1:** Fit indices of latent class solutions.

Latent Class Number	AIC	BIC	Log-Likelihood	Entrophy
2	7575.796	7684.655	1.3074	1.000
3	3714.522	3827.746	3.4735	0.998
4	2872.825	3016.519	5.4392	0.998
5	2315.698	2489.872	5.4235	0.995

**Table 2:** Sample sizes of latent class involvement by gender.

	Boys	Girls
	*N* (%)	*N* (%)
Non-involvement	147 (63.9%)	253 (75.7%)
Psychological–sexual involvement	45 (19.6%)	56 (16.8%)
Psychological–physical involvement	13 (5.7%)	16 (4.8%)
Mild concurrent violence	10 (4.3%)	5 (1.5%)
Severe concurrent involvement	15 (6.5%)	4 (1.2%)

**Table 3:** Impact of quality on being involved in separated violence.

	Quality	Boys Separate Involvement		Girls Separate Involvement	
		β	OR	β	OR
Non-involvement	Companionship	−0.059	0.943	−0.365	0.952
	Intimate communication	−0.402	0.669	−0.049	0.845
	Emotional support	0.205	1.227	−0.168	0.964
	Approval	−0.208	0.812	−0.037	0.968
	Satisfaction	0.235	1.265	−0.033	0.694
	Conflict	0.616 **	1.852	−0.366	1.697
	Criticism	−0.254	0.776	0.529 **	0.486
	Pressure	0.065	1.067	−0.721 **	0.989
	Exclusion	0.083	1.087	−0.011	1.535
	Dominance	−0.008	0.992	0.429	1.364

Note: Reference class: ** *p* < 0.01.

**Table 4:** Regressions between separate involvement and concurrent involvement.

	Quality	Boys Separate Involvement		Girls Separate Involvement	
		β	OR	β	OR
Non-involvement	Companionship	−0.089	0.914	−0.869	0.419
	Intimate communication	0.152	1.165	0.397	1.487
	Emotional support	−0.841 *	0.431	−0.423	0.655
	Approval	0.338	1.402	0.461	1.585
	Satisfaction	−0.018	0.982	−0.539	1.715
	Conflict	0.710 *	0.492	−0.036	1.037
	Criticism	0.482	1.619	0.320	1.377
	Pressure	0.810 *	2.249	−0.202 **	0.817
	Exclusion	−0.121	0.886	−0.117	0.889
	Dominance	−0.200	0.819	0.529	1.697

Note: Reference class: * *p* < 0.05; ** *p* < 0.01.

## Data Availability

The data that support the findings of this study are available from the corresponding author upon reasonable request.
